# Nickel and human sperm quality: a systematic review

**DOI:** 10.1186/s12889-024-21119-y

**Published:** 2024-12-19

**Authors:** Denis Vinnikov, Sergei Syurin

**Affiliations:** 1 al-Farabi Kazakh National University, 71 al-Farabi avenue, Almaty, 050040 Kazakhstan; 2 Peoples’ Friendship University of Russia (RUDN University), 6 Miklukho-Maklaya street, Moscow, 117198 Russian Federation; 3 Northwest Public Health Research Center, 4 2-Sovetskaya street, Saint-Petersburg, 191036 Russian Federation

**Keywords:** Epidemiological, Exposure, Nickel, Review, Bias

## Abstract

**Background:**

Reproductive effects of chronic exposure to nickel (Ni), including sperm quality, have been a matter of debate given that published studies yielded contrasting results. We have, therefore, planned to systematically search and analyze medical literature with the aim to ascertain the association of exposure to nickel with the sperm quality in humans.

**Materials and methods:**

We systematically searched Pubmed, Scopus and Embase for studies reporting the association of Ni with the sperm quality in humans with no time or language limits and used PRISMA to report the findings. The risk of bias was assessed using JBI critical appraisal checklist and SIGN tool. Because the reported effects were no coherent, meta-analysis was not possible.

**Results:**

All included studies were observational and planned to test the effect of a group of trace elements, but not Ni alone. We identified and included 19 studies from 23 publications, published from 12 countries, which assessed sperm quality, sperm DNA damage and sperm metabolome. Ni was quantified in blood, semen plasma, spermatozoa and urine. Sixteen included cross-sectional studies were of acceptable quality, whereas three more case-control reports were of poor quality. Multivariate models were reported in only eight studies. Overall, studies were inconsistent in the direction of effect, when elevated Ni was not associated with the outcome (*N* = 8 studies), or some association was present (*N* = 11 studies). In the latter, 9 studies yielded elevated risk and 2 studies exhibited protective effect. Only one report was in an occupationally exposed population with some association with tail defects, but present in both welders and controls.

**Conclusions:**

Existing evidence from the studies in humans is inconsistent and does not confirm a clear adverse effect of higher Ni concentrations in blood, urine or semen on the sperm quality. Robust methodology must be a key issue in the future studies. Studies with more powerful evidence, such as cohort or experimental reports are needed.

**Supplementary Information:**

The online version contains supplementary material available at 10.1186/s12889-024-21119-y.

## Background

Exposure to heavy metals, including nickel (Ni), is ubiquitous and in humans may occur through inhalational, digestive routes and dermal contact [1, 2]. Ni as metal and in alloys is widespread in the environment, but usually in conjunction with other heavy metals, such as cadmium, resulting in the elevated risk of a wide range of health outcomes in selected occupational groups and in the general population. Occupational exposure is not uncommon. Production of electrical parts, appliances, batteries and accumulators may expose workers to high concentrations of both water-soluble and water-insoluble Ni compounds, which exhibit contrasting toxicity and may trigger specific toxicity mechanisms [3–5]. Therefore, these two groups of nickel compounds have different occupational exposure limits [6]. Ni ore mining, metallic Ni and Ni alloys production have traditionally served as sources of epidemiologic evidence of the health effects of Ni, including its carcinogenic effects [7]. Carcinogenicity of Ni retains most interest as before, but recently other health effects of the occupational exposure to Ni have attracted attention, including pulmonary health outcomes. Recent studies from occupationally exposed Ni industry workers have shed more light to the effects of exposure to Ni dust [8, 9].

Although exposure to Ni in the occupational groups remains most studied, lower levels of exposure in everyday life in the general population raise concerns over its toxicity [10], including carcinogenicity, endocrine disrupting, reproductive, respiratory and cardiovascular effects. One of target organs for Ni is reproductive system, and the evidence on Ni toxicity is traditionally abundant from the animal studies [11–18]. Direct cellular damage, toxic effects with regard to DNA and other mechanisms jointly contribute to Ni toxicity in those animal studies. Reports from humans have also demonstrated adverse effects on human reproduction [19, 20] even with some socio-demographic implications [21]. Moreover, reduced human sperm quality and its lower reproductive potential have been linked with toxic metals, including Ni, in a few in vitro studies [22], whereas the epidemiological evidence from the observational studies remains limited. Those few studies are not consistent in showing negative effects of Ni on human sperm quality.

Such inconsistency may result from the study design, which were cross-sectional in most cases and all were completed in the general population, but not from the Ni industry, in which greater exposure is likely allowing to observe more powerful effects. In addition, selection bias may also explain contrasting effects of these studies, given that most reports were from self-admitted men either treated for infertility or from couples undergoing medical examination for infertility. Moreover, exposure misclassification in the epidemiological studies may be of concern because it remains unclear whether cross-sectional estimation of blood or semen Ni truly represents exposure and how dietary Ni intake should be distinguished from the Ni from other sources, including occupational. Furthermore, published reports have been initially designed to test the effects of multiple trace elements and none of them were specifically planned to verify exposure from this metal. Earlier publications of reproductive Ni toxicity were from welders, where exposure was always mixed, but the study design did not assume to distinguish health effects of welding fumes components.

Medical studies published to-date were not unidirectional in the effects reported, mixed exposure in many of them was likely, and altogether that necessitated a systematic analysis of the medical literature. We have, therefore, planned to systematically search and analyze medical literature with the aim to ascertain the association of exposure to Ni with the sperm quality in humans.

## Materials and methods

### Search strategy

We hereinafter present our analysis according the PRISMA guidelines. We systematically searched Pubmed, Scopus and Embase for entries from their inception to June 2023 in all languages. In Pubmed, we used targeted search of pre-determined keywords in the abstracts and titles with [(nickel) AND (sperm)]. We also used [(nickel) AND (infertility)] search. In Scopus, we used “nickel and sperm”. Finally, we searched for ‘nickel’ AND ‘sperm’ in Embase. Altogether, such search keywords combination in three databases returned 604 items. These items were independently screened by two authors to exclude publications of animal studies and models, all in vitro and cell studies, conference reports and presentations, correspondence, studies which did not report clear outcomes of human sperm quality, as well as studies not directly related to the topic of interest. We thus identified 26 publications, for which we downloaded the full-text versions or requested them from the corresponding authors whenever the latter were not freely available. All these publications were in English, despite we have not applied language filters. These 26 full-text articles were subsequently read by two authors to ensure these papers were conducted in humans, had a clear definition of the aim, exposure and the outcome, and in which the effect was clearly articulated.

**Fig. 1:**
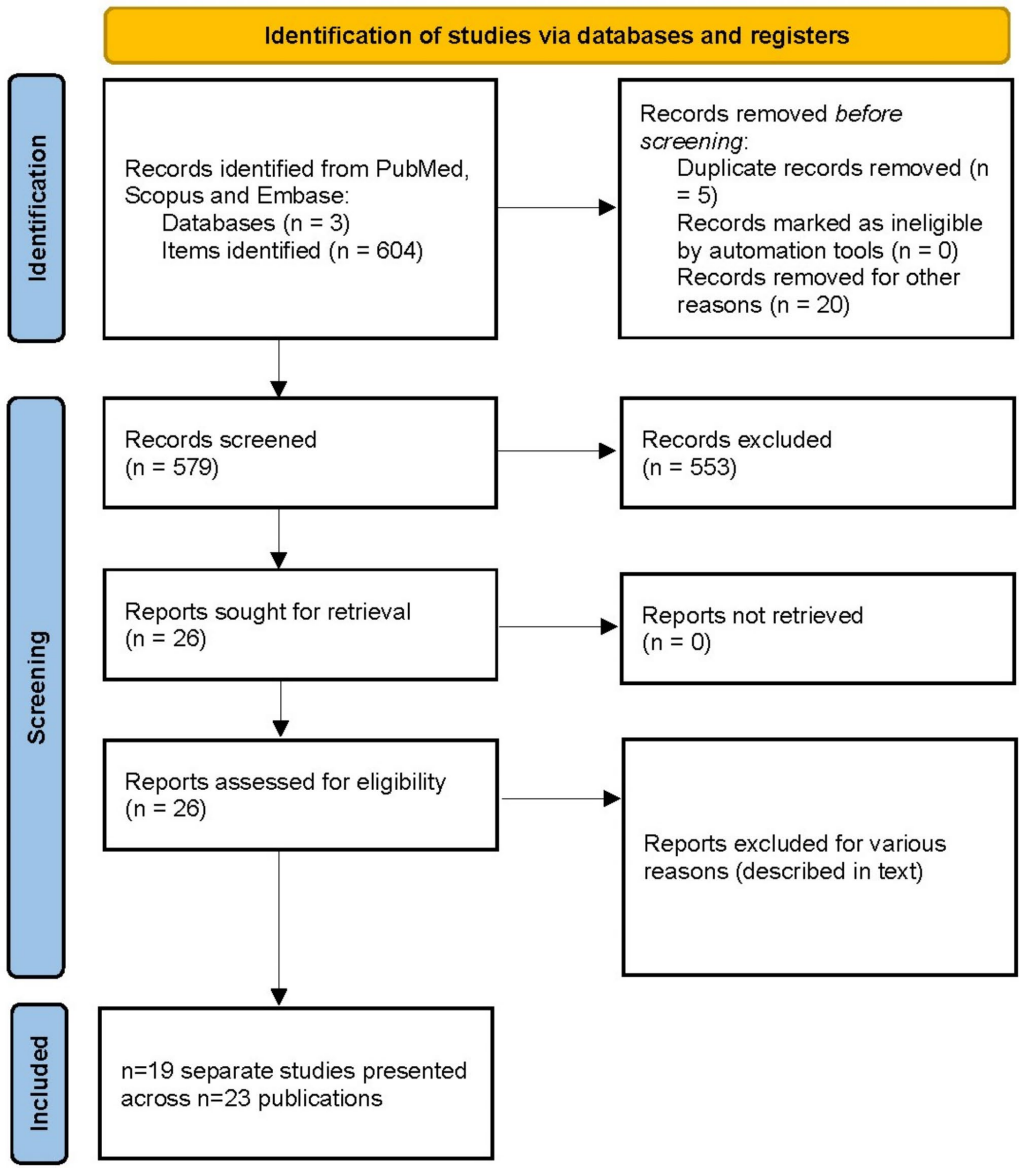
Flow diagram illustrating how studies were screened and selected

We found no duplicate publications. Of those 26 eligible articles, an Italian study [23] was excluded because there were no sperm outcomes and the subsequent associations of blood and sperm trace elements with such outcomes reported in the study. A study of Huang et al. [24] was excluded because it measured Ni and other trace elements in the collected air samples, but not human body liquids. A study of Saglam et al. [25] was excluded because Ni in all studied samples was below the lower limit of detection and thus was not analyzed. Finally, we also cross-checked the references in all 26 eligible papers to search for reports eventually not captured by the search syntax in three included databases we applied. Such search did not identify any other papers, not originally included. Our strategy allowed to eventually include 19 studies, published as 23 papers. The discrepancy between the number of studies and the number of publications arose from five publications of the group of researchers from China, who initially enrolled 1257 subjects and reported various outcomes of the same exposure in papers consecutively published in 2016, 2017, 2019 and 2022 [26–30]. These five publications did not duplicate each other in the outcomes reported, used the same population but reported different sample sizes. Because they all were completed and eventually published from a cohort of 1257 subjects, we have decided to treat those as one large study from five publications. The overall flow of study search, selection and inclusion is presented in Fig. 1.

### Data extraction

Two authors independently extracted data from 23 eventually included publications, where extracted and analyzed information included at least authors, year, country, sample size and its description, study design, exposure classification, outcome classification, the measure of effects and the effect size, as well as whether confounding was considered and how it was addressed. Discrepancies between two independent authors were resolved with discussion and consensus. We performed a targeted data extraction to classify studies into the groups based on the study design. Moreover, we specifically extracted data on how exposure was identified and defined, what outcomes were reported and how the outcome(s) were classified. With regard to the effects, we extracted all reported effects, including absolute and relative measures and even simple two-group comparisons.

### Quality assessment and risk of bias

Because the studies identified in the current report applied cross-sectional and case-control designs, we applied two widely used tools to assess the risk of bias, one for each study guided by the study design. Among a variety of instruments to assess the quality of studies for reviews [31], we have selected JBI critical appraisal checklist for an analytical cross-sectional study [32]. This tool assumes the use of only eight questions and does not assume a quantitative conclusion on the study quality. There is no cut-off level to segregate studies into the ones of high and low quality. We, therefore, report each question of the tool in a designated table to compare studies and report biases. For case-control studies, we used SIGN methodology checklist [31], which allows to group questions on study quality into two sections (eleven questions in section one and four more questions in section two) with the final stratification into ‘high quality’, ‘acceptable’ and ‘low quality’ studies. All studies independent of their quality were included in the review, and their quality as defined by two independent researchers, was summarized in the corresponding tables.

### Statistics and meta-analysis

Despite sufficient number of studies in this review, mathematical pooling of effects in meta-analysis was not possible. Studies reported non-uniformed effects, relative effect measures were either not reported at all (majority of included studies), or confidence intervals were not provided.

## Results

### Overview of included studies

Overall, we identified and included 19 studies from 23 publications [26–30, 33–50] in the current review (Table 1). One study was published as a cohort prospective observation, three case-control studies and the remaining 19 reports were published as cross-sectional studies. A study claimed as a prospective cohort [33], was not purely considered a cohort observation by our group, but a two-stage cross-sectional study instead, since a cohort study by definition assumed that all participants must have been free of a disease (outcome) at entry and thus be at risk for a disease during the follow-up. Therefore, we concluded that the evidence on the association of Ni and sperm quality in humans at present was based on cross-sectional and case-control observational studies.

Altogether, studies were from 12 countries, while analyses from the Chinese population were the most prevalent (7 of 19 studies, 37%). Only three of included 23 studies (13%) were published more than 10 years ago, reflecting dramatic increase in the interest to the topic only recently. The total number of participants in all studies was 4640, and the majority of included subjects (*N* = 3653, 79%) were from China. All but one study was completed in the young or middle-aged men. Most studies were designed and implemented in men visiting reproduction centers as either healthy subjects undergoing examination with their partners for infertility or as patients already with the diagnosis of infertility.

**Table 1:** Summary of included studies

#	Authors, ref	Country, year	Sample, expected source of exposure	Exposure classification	Outcomes	Effect
1	Jia et al., [34]	China, 2022	*N* = 841, visitors of the Reproduction Center, age 29.6 ± 5.5 years. Environmental/unspecified	Ni in seminal plasma using ICPMS	21 parameters, of which 5 (semen volume, sperm concentration, total sperm count, progressive motility, and normal morphology)	In adjusted for smoking status, age, alcohol intake status, and BMI model, Ni-60 was associated with reduced semen quality (OR 1.52 (1.14, 2.03)); in adjusted for model 1 + all other metals, OR was 1.30 (1.08, 1.56).
2	Chai et al., [33]	China, 2022	*N* = 666 followed up after one year (*N* = 796 included at baseline), college students, mean age 20 years at baseline. Environmental/unspecified	Ni in urine using ICPMS (creatinine-adjusted) (2 samples)	Sperm concentration, total motility, progressive motility, sperm count and volume	Ni was associated with a lower rate of normal sperm morphology rate (adjusted for other elements, age, abstinence time, BMI, tobacco smoking, alcohol consumption and tea drinking beta − 4.28 (-7.14;-1.33)).
3	Karabulut et al., [35]	Turkey, 2022	*N* = 60 with normozoospermia and *N* = 53 with at least one abnormal semen parameter, age 18–52, all visitors of the reproduction center. Environmental/unspecified	Ni in seminal plasma using ICPMS	Sperm concentration, total motility, progressive motility, sperm count and volume	No difference in Ni seminal concentration between normozoospermic (5.74 ± 10.18 ng/ml) and the alternative (7.07 ± 14.43 ng/ml) groups.
4	Shi et al., [36]	China, 2021	*N* = 288 visitors of the andrology laboratory, age 37.9 ± 5.4 years. Environmental/unspecified	Ni in blood using ICPMS	Sperm concentration, motility and morphology, sperm vitality, in vitro sperm capacitation and acrosome reaction assay, sperm DNA fragmentation	Blood Ni concentration was not associated with any parameter studied in the multivariate models.
5	Calogero et al., [37]	Italy, 2021	Volunteers from the general sample of an industrial and agricultural areas, *N* = 179, mean age 32 ± 6 years. Environmental/unspecified	Ni in (1) seminal plasma; (2) venous blood; and (3) spermatozoa using ICPMS	Sperm concentration, total sperm count, progressive motility, and normal forms	No effect on seminal plasma or spertamotozoa. Higher blood Ni was in those with lower sperm concentration (12.30 vs. 7.69 µg/L), with lower total sperm count (19.07 vs. 7.04 µg/L), with lower progressive motility (14.50 vs. 2.75 µg/L).
6	Rodriguez-Dias et al., [38]	Canary Islands, 2021	Visitors of the Reproduction Center, *N* = 102, age 38.0 ± 5.7. Environmental/unspecified	Ni in seminal plasma using ICPMS	Sperm concentration and motility	Seminal Ni was not statistically different between patients with normal sperm (0.042 ± 0.191 mg/kg) and sperm with pathology (0.025 ± 0.072 mg/kg).
7	Jewad et al., [39]	Iraq, 2019	Visitors of the Reproduction Center, *N* = 66 cases of infertile men, age 27–31 and *N* = 30 controls, age 17–40. Environmental/unspecified	Ni in seminal plasma using furnace atomic absorption spectrometry	Sperm concentration, total sperm count, morphology, motility grades	Seminal Ni concentration was significantly different in fertile men (25.55 ± 4.01 pbb) when compared to primary infertile men (18.45 ± 4.05 ppb) and secondary interfile men (20.26 ± 4.96 ppb).
8	Bian et al., [40]	China, 2019	Volunteers, *N* = 205, age 20–50 years. Environmental/unspecified	Ni in seminal plasma using ICPMS	Sperm concentration, total sperm count and progressive motility	Higher seminal plasma Ni-58 was in the group of samples with better motility (more than 40%) (10.22 ± 3.83 vs. 5.69 ± 1.93 µg/L). Higher seminal plasma Ni-60 was in the group of samples with better motility (more than 40%) (10.75 ± 3.86 vs. 6.55 ± 2.11 µg/L).
9	Jain et al., [41]	India, 2016	*N* = 13 males with spermiation defect (age 31.7 ± 4.5) and 20 normal fertility controls (age 28.3 ± 6.8 years). Environmental/unspecified	Ni in the seminal cells/seminal debris using scanning electronic microscope. Ni in (1) serum and (2) seminal plasma using ICPMS	Azoorspermia (spermiation defect)	Serum Ni in patients with spermiation defect was 6.3 ± 3.7 ppb compared to 1.61 ± 1.18 ppb in controls. Ni in seminal plasma was 5.41 ± 1.42 ppb in cases with spermiation defect and 5.35 ± 10.54 ppb in controls.
10	Zhou et al., [42]	China, 2016	Infertile men, visiting Reproduction Center, *N* = 207. Environmental/unspecified	Ni in urine using ICPMS (creatinine-adjusted)	Comet assay parameters (DNA damage) including tail length, percent DNA tail, and tail distributed moment	4th quartile of urinary Ni was associated with tail length (beta 2.95; 95% CI 0.34;5.56) adjusted for all included trace elements, age, BMI, abstinence time and smoking.
11, 12, 13, 14, 15	Wang et al., [26] Wang, et al., [27] Wang et al., [28] Wan et al., [29] Xu et al., [30]	China, 2016, 2017, 2019, 2022	Visitors of the Reproduction Center, *N* = 1052 (*N* = 1247 screened), age 32.1 ± 5.4. Subjects with occupational exposure to metals excluded. Environmental/unspecified	Ni in urine using ICPMS (creatinine-adjusted) (2 samples), additionally Ni in seminal plasma (Wan et al., Xu et al.)	Sperm concentration, total sperm count, progressive and non-progressive motility, comet assay an Annexin V assay, CASA motion parameters (straight-line velocity, curvilinear velocity, average path velocity, linearity, straightness and amplitude head displacement. Seminal plasma metabolome	Adjusted for smoking and creatinine, urine Ni was associated with reduced total motility (beta coefficient for a 10-fold increase in the ln-transformed mean urinary Ni -2.90 (95% CI -5.66;-0.09). Adjusted for age, BMI, abstinence time, alcohol use, smoking status and urinary creatinine, Ni was significantly associated with PI + spermatozoa and Annexin V-/PI- spermatozoa. In a fully-adjusted model, neither urinary Ni, nor seminal plasma Ni was associated with any CASA parameter. Ni was not associated with any studied metabolic marker in the seminal plasma.
16	Skalnaya et al., [43]	Russian Federation, 2015	Visitors to the commercial laboratory, *N* = 148. Environmental/unspecified	Ni in semen using ICPMS	Ejaculate volume, sperm count, sperm motility, and vitality	Total sperm count, relative sperm count, sperm motility and sperm vitality were not associated with Ni concentration in ejaculate.
17	Zafar et al., [44]	Pakistan, 2015	Visitors to the Reproduction Center, *N* = 75. Environmental/unspecified	Ni in seminal plasma using ICPMS	Sperm concentration, total sperm count, motility	There was a significant difference in seminal plasma Ni concentrations between normozoospermia (3.07 ± 1.63 ppb), oligozoospermia (1.92 ± 0.77 ppb) and azoospermia (10.49 ± 10.94 ppb) groups. The effect was likely mixed with Cd.
18	Zeng et al., [45]	China, 2015	Visitors to the infertility, *N* = 394, age 31.4 ± 5.5 years. Environmental/unspecified	Ni in urine (creatine-adjusted) using ICPMS	Sperm concentration, count, motility, normal morphology and abnormal head	Ni was not associated with sperm concentration, motility or count, but a significant association was found for % of abnormal head; men in the second quartile of Ni had a significant increase in sperm normal morphology of 2.02% (95% CI: 0.14, 3.90).
19	Guzikowski et al., [46]	Poland, 2015	Infertile men, *N* = 34, mean age 28.9 years. Environmental/unspecified	Ni in seminal plasma using ICPMS	Sperm count, sperm motility and sperm morphology	No correlation was found between Ni in seminal plasma with sperm count, sperm motility and sperm morphology.
20	Schmid et al., [47]	USA, 2013	*N* = 20 healthy male volunteers, including 10 men aged 22–28 and 10 men aged 65–80 years. Environmental/unspecified	Ni in seminal plasma and washed sperm using proton-induced X-ray emission	Sperm count and motility. Comet assay	In the adjusted models the association was not found.
21	Slivkova et al., [48]	Slovak Republic, 2009	*N* = 47, visitors to the infertility center, age 22–48. Environmental/unspecified	Ni in semen using flame absorption spectrophotometry	Spermatozoa microscopy	No association was identified.
22	Danadevi et al., [49]	India, 2003	*N* = 28 welders (age 32.3 ± 4.4 years) and *N* = 27 (age 32.2 ± 4.7 years) unexposed controls. Occupational/environmental	Ni in blood using ICPMS	Sperm count, motility and morphology	Blood Ni was significantly associated only with tail defects in both welders (beta 0.422) and controls (beta 0.485), but not with sperm count, motility, head defects or vitality.
23	Umeyama et al., [50]	Japan, 1986	*N* = 22 fertile men, age 32.5 ± 3.4 and *N* = 69 infertile men, age 33.4 ± 4.3 years. Environmental/unspecified	Ni in semen using ICPMS	Unclear. Cases were classified into normozoospermic, oligozoospermic, severe oligozoospermic and azoospermic	There was a non-significant higher Ni concentration in fertile men (0.080 ± 0.033 mg/L) compared to infertile men (0.066 ± 0.041 mg/L).

*Note: ICPMS - Inductively Coupled Plasma Mass Spectrometry; CASA – computer-assisted sperm analysis*

None of published studies were specifically designed to ascertain the effect of Ni only, but in all cases analyzed a group of trace metals. Inductively coupled plasma mass spectrometry (ICPMS) was a dominating analytical method in the majority of included reports, both in newer and older studies. In most publications, subjects were questioned on their occupational status and those with known exposure to Ni in the workplace were excluded. There was, however, a study completed in welders comparing them with controls with known exposure to Ni verified with measured Ni concentration in blood [49]. Therefore, most reports dealt with relatively low Ni concentrations, presumably from environmental exposures. Out of 19 included studies Ni was measured in blood in four studies (21%) [36, 37, 41, 49]; in the urine in five studies (26%) [26–30, 33, 42, 45]; in seminal plasma or sperm in fourteen studies (74%) [29, 30, 34, 35, 37–41, 43, 44, 46–48, 50]; and finally in spermatozoa in 2 studies (11%) [37, 41].

As for the outcome, the majority of studies reported conventional sperm morphology, including computer-assisted tests, and patients were divided into those with normal and abnormal sperm based on this sperm analysis in most studies. In addition, four studies characterized comet assay (DNA damage) [27, 28, 36, 42, 47] and one more study reported seminal plasma metabolome [30]. The association of Ni with one or more outcomes was reported in each included study. Univariate comparisons of Ni concentrations with the selected outcome were presented in eleven studies, where Ni was compared between two or more groups. Adjusted models were reported in eight studies [26–30, 33, 34, 36, 37, 42, 45, 47], where the authors offered a non-uniform set of confounders, which could differ between studies. The set of variables for such adjusted models was dictated by the first step of the univariate comparisons, preceding research and even biological plausibility in selected studies.

When all studies were analyzed altogether, there was no consistency in the effects of Ni across these studies. Overall, there was some association of Ni with the outcome in eleven studies, whereas in eight studies the effect was not confirmed. In the group of 11 studies with some effect, higher Ni concentrations were associated with poorer or adverse outcomes in nine studies, whereas the remaining two studies showed the opposite effect. Table 2 illustrates inconsistency in the direction of effect from the included studies. Thus, all four studies of Ni in the urine confirmed higher risk of adverse effects. However, seminal plasma or sperm studies exhibited both risk and protective effects, but most studies in this group demonstrated no association of seminal Ni with the studied outcomes. As Table 2 shows, the protective effect of Ni was only found in studies of seminal plasma or sperm, while urine studies consistently confirmed some negative effect of higher Ni concentrations.

**Table 2:** Stratification of studies in the groups of reported effects

Higher risk effect	No effect	Protective effect
**Studies of Ni in blood (*N*** **= 4)**		
Calogero et al.	Shi et al.	
Jain et al.		
Danadevi et al.		
**Studies of Ni in the urine (*N*** **= 4)**		
Chai et al.		
Zhou et al.		
Zeng et al.		
Wang et al. (1), Wang et al. (2), Wang et al. (3), Wan et al., Xu et al.		
**Studies of Ni in seminal plasma or sperm (*N*** **= 14)**		
Jia et al.	Karabulut et al.	Jewad et al.
Zafar et al.	Calogero et al.	Bian et al.
	Rodriguez-Dias et al.	
	Jain et al.	
	Wang et al. (1), Wang et al. (2), Wang et al. (3), Wan et al., Xu et al.	
	Skalnaya et al.	
	Guzikowski et al.	
	Schmid et al.	
	Slivkova et al.	
	Umeyama et al.	

Our further stratification into the reports of adjusted analyses vs. those with simple univariate comparisons did not clarify the source of inconsistency. Thus, among eight studies with adjusted models, six studies reported elevated risk, whereas the remaining two presentations two did not show such effect. Similarly, the pool of studies with simple univariate comparisons contained reports with no effect, some negative effect and some protective effect of Ni. Of note, studies with protective effect did not imply any adjustment and presented only univariate comparisons. The heterogeneity and the opposite direction of effect persisted even when studies were stratified into those published from China vs. non-Chinese studies.

Of the wide range of outcomes studied, sperm motility, count and concentration were the most prevalent. When only these studies were considered, the direction of effect was again wide enough from no effect to higher risk of abnormal motility, count or concentration. Furthermore, two studies in the current review elucidated the protective effect of Ni on the sperm motility, count and concentration. Therefore, studies reporting conventional routine sperm analysis failed to confirm the association of higher Ni with poorer sperm outcomes.

### Assessment of the risk of bias

Two identified study designs in this review implied the use of two tools for quality assessment. Eight questions of JBI critical appraisal checklist for cross-sectional studies are summarized in Table 3 highlighting the strengths and limitations of each publication. Such critical appraisal showed that, overall, cross-sectional studies were of acceptable quality, when 7 out of 16 (48%) cross-sectional studies had all ‘yes’ answers, thus being of the high quality, clearly identifying samples, classifying exposure and the outcome, as well as addressing confounding. Samples or inclusion/exclusion criteria were not clearly defined in eight studies. Our critical appraisal demonstrated that the exposure and the outcomes were properly identified and described in most of the studies (Table 3). The major problem in the included 16 studies was confounding and the way the authors dealt with it. There was a trend of ignoring confounding in earlier studies, whereas most recent reports identified potential variables and included them in the multivariate comparisons. We also found that studies will smaller samples were more likely to report unadjusted effects when confounding was not considered. Finally, most studies from China clearly demonstrated a robust approach to select and address confounding in their multivariate models.

**Table 3:** Summary data of the quality assessment in the included studies

1. Cross-sectional studies								
	Criteria for inclusion clearly defined	Study subjects and setting described	Exposure measured in a valid way	Standard criteria used for measurement	Confounders identified	Strategies to deal with confounders stated	Outcomes measured in a valid way	Appropriate statistical analysis used
Jia et al., 2022	Y	Y	Y	Y	Y	Y	Y	U
Chai et al., 2022	Y	Y	Y	Y	Y	Y	Y	Y
Karabulut et al., 2022	Y	N	Y	Y	N	N	U	Y
Shi et al., 2021	Y	Y	Y	Y	Y	Y	Y	Y
Calogero et al., 2021	Y	Y	Y	Y	Y	Y	Y	Y
Rodriguez-Dias et al., 2021	Y	N	Y	Y	N	N	Y	Y
Bian et al., 2019	N	U	Y	Y	N	N	Y	U
Zhou et al., 2016	Y	Y	Y	Y	Y	Y	Y	Y
Wang et al., Wang, et al., Wang et al., Wan et al., Xu et al., 2016, 2017, 2019, 2022	Y	Y	Y	Y	Y	Y	Y	Y
Skalnaya et al., 2015	N	N	U	Y	N	N	Y	Y
Zafar et al., 2015	N	N	Y	Y	N	N	Y	Y
Zeng et al., 2015	Y	Y	Y	Y	Y	Y	Y	Y
Guzikowski et al., 2015	Y	N	Y	Y	N	N	Y	Y
Schmid et al., 2013	Y	Y	Y	Y	Y	Y	Y	Y
Slivkova et al., 2009	N	N	Y	U	N	N	Y	Y
Danadevi et al., 2003	N	N	Y	Y	N	N	Y	Y
2. Case-control studies								
						Jewad et al., 2019 [39]	Jain et al. [41]	Umeyama et al., [50] 1986
1.1 The study addresses an appropriate and clearly focused question						Y	Y	Y
1.2 The cases and controls are taken from comparable populations						U	N	N
1.3 The same exclusion criteria are used for both cases and controls						U	U	U
1.4 What percentage of each group (cases and controls) participated in the study?						U	U	U
1.5 Comparison is made between participants and non-participants to establish their similarities or differences						N	N	N
1.6 Cases are clearly defined and differentiated from controls						N	Y	N
1.7 It is clearly established that controls are non-cases						N	Y	Y
1.8 Measures will have been taken to prevent knowledge of primary exposure influencing case ascertainment						U	U	U
1.9 Exposure status is measured in a standard, valid and reliable way						U	Y	Y
1.10 The main potential confounders are identified and taken into account in the design and analysis						N	N	N
1.11 Confidence intervals are provided						N	N	N
Overall study rating						0	0	0

*Note: Appraisal of cross-sectional studies was completed with JBI critical appraisal checklist; Y – yes; N – no; U – unclear. Appraisal of case-control studies was completed with SIGN checklist*

Case-control studies (*N* = 3) were all of low quality (Table 2), because we found significant flaws in study design, ascertainment of cases and controls and the way confounding was managed. We also found that in all studies the risk of bias was high, given that selection of cases and controls was unclear (selection bias) and no clear approach was utilized to address confounding. None of included case-control studies mentioned confounding. None of included studies provided any sample size calculation based on the known effect and ratio of cases to controls. The ratio itself has not been justified. As Table 2 shows, all case-control studies only compared Ni concentrations between groups in the univariate comparisons with no relative measures of effect.

### Synthesis

A number of small and moderate-size observational studies, which employed only cross-sectional and case-control designs was published. Cumulative evidence from these cross-sectional studies of satisfactory quality and a few more case-control studies of low quality does not allow to conclude higher risk of adverse sperm outcomes in subjects with greater exposure to Ni as measured via Ni concentrations in blood, urine or semen. Inconsistency in the direction of effect persisted when studies were further stratified into those reporting univariate comparisons vs. adjusted analyses. Furthermore, the contribution of confounding was likely high, given that the sources of exposure may be very diverse and may include both everyday environmental sources and the workplace. In addition, most included studies were accomplished in the environmentally exposed population, but not occupational groups, and the level of exposure in the former may be quite low to allow for the detectable effect. Moreover, most published studies in the world literature and identified in the current review employed cross-sectional design with limited or no potential to verify causality. In such context, finding higher levels of Ni in the seminal plasma does not mean that Ni is the reason for sperm abnormality. Taken together, conclusions on the robust association of higher Ni concentrations in the semen plasma, blood or urine are preliminary and should be further explored in prospective observations and experimental controlled studies. Meta-analysis pooling of effects was not possible.

## Discussion

Exposure to Ni, a ubiquitous heavy metal, has been a subject of growing concern due to its potential adverse health effects. In this review, we systematically analyzed 19 studies to ascertain the association between Ni exposure and sperm quality in humans. The studies included in our analysis were primarily cross-sectional and case-control observational studies, which provided insights into the effects of Ni on sperm parameters. In general, we found that evidence on the adverse effect of Ni on sperm quality is insufficient, studies lack scientific rigor, and one of the major concerns is confounding.

Exposure to Ni may be both work-related [51] and environmental [52]. The latter, including digestion from water, inhalation and dietary intake, may yield overall lower level of exposure [53], but it remains unclear whether relatively low environmental levels of exposure may be associated with clinically meaningful effects [54], including reproductive outcomes. When such exposure is mixed with some occupational inhalation of Ni-containing dust [55], clinical effects may manifest with some respiratory, endocrine and reproductive conditions, and the current review could shed more light onto the true association of exposure to Ni with one of such outcomes. It is important to note that the included studies were primarily conducted in environmentally exposed populations, rather than occupational groups with higher levels of Ni exposure. This distinction is crucial since the level of exposure in the general population may be too low to detect significant effects on sperm quality. Furthermore, most studies employed cross-sectional designs, which are limited in their ability to establish causality and infer the temporal relationship between Ni exposure and sperm outcomes.

One of the reasons of such inconsistency could be an ongoing debate whether Ni in blood, urine or sperm better reflects exposure. Some studies suggest analyzing Ni concentrations in urine, while others advocate for blood measurements. This discrepancy reflects the complex nature of Ni exposure, which can occur through various routes such as inhalation, ingestion, and dermal contact. The different exposure routes may lead to variations in the distribution and accumulation of Ni in different bodily compartments [56, 57], making it challenging to determine the most representative biomarker for assessing exposure. A few studies also included in this review discussed whether urine Ni concentrations could serve as a better and more stable marker of exposure [26, 27, 45]; however, Ni is still largely measured in blood and seminal plasma, and the current review demonstrated that seminal plasma was most often used to ascertain exposure to Ni. Our analysis also revealed that all studies investigating Ni concentrations in urine consistently demonstrated a higher risk of adverse effects with increasing Ni levels. In contrast, studies focusing on seminal plasma or sperm demonstrated both risk and protective effects, with the majority failing to find a significant association between Ni concentrations and the studied outcomes. These inconsistent findings suggest that the effects of Ni on sperm quality may vary depending on the specific compartment or biological fluid analyzed.

Confounding remains a significant concern in studies assessing the association between Ni exposure and sperm quality. The sources of exposure to Ni are diverse, including those of both environmental and occupational origin, making it challenging to purify the effects of Ni alone. No adverse effect of Ni in some studies even when known confounding is considered, as this review demonstrates, may indicate that the relationship of Ni with sperm quality may be more complex than previous animal models have shown. Sperm quality may be associated with a very large range of potential confounders, and a few known risk factors for sperm abnormality were considered in the included original studies, when patients with varicocele, epididymitis and other conditions were excluded. However, recent studies have now revealed other previously underrecognized conditions, which may worsen sperm quality, and these include obesity [58], diets rich in saturated fatty acids and low in polyunsaturated fatty acids [59], bisphenol-A [60, 61] and many other conditions and lifestyle attributes. Another source of unmeasured confounding in the included studies was apparently the use of supplements, which is hard to verify in observational studies [62]. Moreover, in many included studies well-studied risk factors such as diabetes [63], tobacco smoking [64, 65], alcohol use [66] were not controlled for. We believe that confounding acted as the most meaningful contributor to heterogeneity of studies and a non-uniform direction of effect. Future studies should aim to address confounding more comprehensively by considering potential confounders, such as lifestyle factors, occupational history, and other co-exposures, in their analytical models.

Finally, in some included studies Ni effects were assessed not with regard to sperm quality, but instead with male infertility [39], and this should be considered when interpreting this review findings as another limitation. In addition, the impact of a single trace element on spermiation may differ from the combined effects of multiple trace elements. The outcome can vary based on how the elements interact, their specific roles in the spermiation process, and their concentrations. These differences arise from the potential for synergistic, antagonistic, or additive interactions among the elements; therefore, future studies should be planned with stricter exposure classification and a way to foresee the interaction of trace element when assessing the association with health outcomes.

The findings of this analysis should be interpreted with caution given that comparing healthy participants with diagnosed infertile participants may exaggerate the effect of nickel on sperm. This occurred because most studies were initially designed and implemented not as population-based, but as studies on men admitted to the Reproduction Centers for infertility and thus selection bias was likely. Future studies should consider population-based sampling to reduce this selection bias.

Overall, the current evidence on the association between Ni exposure and sperm quality in humans is limited and inconsistent. The heterogeneity in study designs, exposure assessment, outcome measurement, and the inclusion of confounding factors contributes to the conflicting findings observed across studies. Therefore, it is necessary to conduct further research, including prospective observational studies and well-controlled experimental studies, to better understand the potential effects of Ni on sperm quality and to establish a clearer causal relationship.

## Conclusions

In conclusion, based on the available evidence, our analysis suggests that the association between Ni exposure and sperm quality in humans is complex and inconclusive. While some studies suggest a higher risk of adverse effects with increased Ni exposure, others fail to confirm such associations. The conflicting results underscore the need for further investigation and emphasize the importance of considering confounding factors in future studies. A comprehensive understanding of the potential health effects of Ni exposure on sperm quality will contribute to informed decision-making and the development of preventive strategies in occupational and environmental settings.

## Electronic supplementary material

Below is the link to the electronic supplementary material.

Supplementary Material 1

## Data Availability

All data generated or analysed during this study are included in this published article.

## Abbreviations

CASA: Computer–assisted sperm analysis
ICPMS: Inductively coupled plasma mass spectrometry
Ni: Nickel
